# Transferable Extended-Spectrum β-Lactamase (ESBL) Plasmids in *Enterobacteriaceae* from Irrigation Water

**DOI:** 10.3390/microorganisms8070978

**Published:** 2020-06-30

**Authors:** Maria-Theresia Gekenidis, Anita Kläui, Kornelia Smalla, David Drissner

**Affiliations:** 1 Microbiological Food Safety, Agroscope, 8820 Wädenswil, Switzerland; maria.stergiou@agroscope.admin.ch; 2 Microbiological Food Safety, Agroscope, 3003 Liebefeld, Switzerland; anita.klaeui@agroscope.admin.ch; 3 Institute for Epidemiology and Pathogen Diagnostics, Julius Kühn-Institut—Federal Research Centre for Cultivated Plants (JKI), 38104 Braunschweig, Germany; kornelia.smalla@julius-kuehn.de; 4 Department of Life Sciences, Albstadt-Sigmaringen University, 72488 Sigmaringen, Germany

**Keywords:** irrigation water, antibiotic resistance, ESBL *Enterobacteriaceae*, conjugative plasmids

## Abstract

Extended-spectrum β-lactamase (ESBL)-producing *Enterobacteriaceae* are classified as serious threats to human health by the U.S. Centers for Disease Control and Prevention. Water used for irrigation of fresh produce can transmit such resistant bacteria directly to edible plant parts. We screened ESBL-producing *Escherichia coli*, *Enterobacter cloacae*, and *Citrobacter freundii* isolated from irrigation water for their potential to transmit resistance to antibiotic-susceptible *E. coli*. All strains were genome-sequenced and tested in vitro for transmission of resistance to third-generation cephalosporins on solid agar as well as in liquid culture. Of the 19 screened isolates, five ESBL-producing *E. coli* were able to transfer resistance with different efficiency to susceptible recipient *E. coli*. Transconjugant strains were sequenced for detection of transferred antibiotic resistance genes (ARGs) and compared to the known ARG pattern of their respective donors. Additionally, phenotypic resistance patterns were obtained for both transconjugant and corresponding donor strains, confirming ESBL-producing phenotypes of all obtained transconjugants.

## 1. Introduction

The discovery and subsequent broad use of antibiotics in the first half of the last century has revolutionized human and veterinarian medicine. However, a setback promptly followed, as upon discovery of each novel antibiotic, emergence of resistant bacteria soon followed [1]. Antibiotic resistance is conferred by three mechanisms: (1) bacterial target alteration, e.g., by mutation, (2) drug inactivation, e.g., by hydrolyzing enzymes, and (3) decreased intracellular drug concentration through low cell permeability or drug efflux pumps [2]. The genes conferring such antibiotic resistance (ARGs) are of special concern to human health when carried by mobile elements such as phages or plasmids, as these can spread rapidly within bacterial communities and, thus, from commensal or environmental bacteria to human pathogens [3]. The extent and speed of this so-called horizontal gene transfer (HGT) depends on various factors such as bacterial density (biofilms vs. planktonic cells), conditions supporting bacterial growth, or plasmid type (broad- or narrow-host-range plasmids). Barriers to HGT exist, such as recipient helicase compatibility or host restriction system susceptibility, which can reduce but not completely prevent the transfer and establishment of a transferable plasmid in the bacterial host cell [4]. Of note, antibiotic resistance (AR) plasmids usually carry several ARGs, often conferring resistance to multiple antibiotic classes. Antibiotic-resistant bacteria (ARB) with acquired resistance to at least one agent in three or more antibiotic classes are referred to as multidrug-resistant (MDR) [5].

ARB can be classified into different threat groups—concerning, serious, and urgent threats—based on the burden they pose for human health. Treatment options for these ARB become more and more limited, leaving only a few last-resort antibiotics at choice for serious and urgent threat ARB. Extended-spectrum β-lactamase (ESBL)-producing *Enterobacteriaceae* are listed by the U.S. Centers for Disease Control and Prevention (CDC) among the serious threats [6]. In its 2019 antibiotic resistance threat report, the CDC counted more than 2.8 million antibiotic-resistant infections causing 35,000 deaths in the United States, and they observed an increase by 50% in infections caused by ESBL-producing *Enterobacteriaceae*. These encompassed ESBL-producing *Escherichia coli* (*E. coli*), *Enterobacter* spp., *Citrobacter* spp., and *Klebsiella pneumoniae*.

The β-lactamase enzyme family can be subdivided into four groups based on protein homology, known as Ambler scheme classes A to D [7]. Extended-spectrum β-lactamases almost exclusively belong to Ambler class A and can be defined as enzymes conferring resistance to penicillins, first- to third-generation cephalosporins, and aztreonam [8]. ESBL-encoding genes are often located on transferable plasmids and can thus spread among bacteria. Such genes are far from confined to clinical settings, and they have also been detected in bacteria from companion and food-producing animals [9,10], in various foods including raw fresh produce [11,12,13,14], as well as different environmental sources such as surface waters [15,16] or agricultural as well as undisturbed soils [17,18]. One of the most important ESBL enzyme families is the CTX-M family [19]. They are assumed to originate from chromosomal *bla*_CTX-M_-related genes from different environmental *Kluyvera* spp. In this process, the involvement of mobile genetic elements (MGEs) such as insertion sequences, class 1 integrons, and transposons has been crucial [20].

Foods such as fresh produce that are usually consumed raw can easily transfer their bacterial communities to the consumer, and many studies have documented the presence of ARB in such products [21,22,23,24]. Fresh produce is often exposed to a broad variety of potential contamination sources, especially when cultured in open greenhouses or fields [25]. One contamination source is surface water such as rivers, channels, or lakes that are often used for irrigation, especially in areas with limited water resources [26]. Additionally, wastewater treatment plants often release treated wastewater into the nearby streams and have been identified as point sources of micropollutants such as antibiotics, ARB, and ARGs into the environment [27]. By irrigating fresh produce, especially overhead, edible plant parts can get contaminated with these micropollutants. Moreover, uptake of pharmaceutical pollutants such as antibiotics by different crops has been shown in real agricultural systems [28], and the introduction of ARB can affect the plant microbiome, which represents a major pathway of human exposure to microbes [29]. Nevertheless, irrigation water is not monitored routinely for pathogenic bacteria, let alone for ARB or ARG content. In Switzerland, a risk analysis for irrigation water has been proposed by SwissGAP since 2017, including reference values for maximal loads of the fecal indicators *E. coli* and *Enterococcus* spp. [30]. Only in case of operational changes on the farm, the risk analysis must be updated. This, however, is not comparable to systematic monitoring. In a previous study, we found that a large proportion (22%) of irrigation water from major Swiss vegetable growing areas contained ESBL-producing *E. coli* [15]. In the present study, we investigated the ability of these ESBL-producing *E. coli* as well as isolates belonging to ESBL-producing *Enterobacter cloacae* (*E. cloacae*) and *Citrobacter freundii* (*C. freundii*) complexes from the same irrigation water sources for their ability to transfer their ESBL-resistance phenotype to a β-lactam susceptible *E. coli*. We conclude that 5 of the investigated 11 ESBL-producing *E. coli* were able to transfer ESBL-encoding genes and ESBL-phenotype by conjugation in both solid and liquid medium experiments, while no conjugation was observed for the ESBL-producing *E. cloacae* or *C. freundii* complex isolates.

## 2. Materials and Methods

### 2.1. Bacterial Strains and Culture Conditions

ESBL-producing *Enterobacteriaceae* were isolated from irrigation water originating from important Swiss vegetable growing areas [15]. Irrigation water sources included groundwater as well as various surface waters such as rivers, creeks, or ponds. Briefly, ESBL-producing *Enterobacteriaceae* were isolated by bacterial enrichment in EE broth Mossel (Beckton Dickinson, Franklin Lakes, NJ, USA) and subsequent streaking onto commercial, ready-to-use Brilliance ESBL plates (Oxoid Ltd., Hampshire, UK). Single colonies with morphologies corresponding to the target bacteria ESBL-producing *E. coli*, *Enterobacter* spp., or *Citrobacter* spp. according to the manufacturer were identified at species level by MALDI biotyping (see Section 2.2), re-grown in lysogeny broth (LB; Sigma-Aldrich, St. Louis, MO, USA) containing ceftazidime (CAZ, 8 or 0.8 mg/L; Sigma-Aldrich), and stored with glycerol at −80 °C. Strains for conjugation experiments and antibiotic susceptibility testing were re-grown in LB with the appropriate CAZ concentration. Representative strains from each bacterial genus were selected for conjugation experiments: 11 ESBL-producing *E. coli* strains (H2, H6, H10, H17, H22, H25, H30, H38, H40, H44, and H45), 5 *E. cloacae* complex strains (H9, H16, H24, H34, and H36), and 3 *C. freundii* complex strains (H12, H41, and H42).

Kanamycin (KAN)- and rifampin (RIF)-resistant, green fluorescent protein (*gfp*)-tagged *E. coli* CV601 [31], serving as a recipient strain in conjugation experiments, was grown in LB containing 50 mg/L of each KAN and RIF.

### 2.2. MALDI Biotyping

MALDI biotyping was performed by direct smearing as described previously [32] using a microflex LT MALDI-TOF mass spectrometer (Bruker Daltonics, Bremen, Germany) and the associated MALDI biotyper RTC Software (Version 3.1; Bruker Daltonics). Notably, species belonging to either *E. cloacae* complex (such as *E. ludwigii* or *E. asburiae*) or *C. freundii* complex (such as *C. braakii* or *C. gillenii*) could not be distinguished according to the manufacturer and were grouped therefore into the corresponding complex.

### 2.3. Antibiotic Susceptibility Testing

For all 19 ESBL-producing *Enterobacteriaceae* from irrigation water, resistance to 32 clinically relevant antibiotics was determined in disk diffusion assays as described previously [15]. Additionally, all ESBL-producing donor and transconjugant strains obtained from filter conjugation experiments were screened for resistance against 31 microbial agents by broth dilution using the MicroScan autoSCAN-4 System (Beckman Coulter Life Sciences, Indianapolis, IN, USA) with the Neg MIC 44 panel. Susceptibility testing was performed according to the manufacturer’s guidelines.

### 2.4. DNA Extractions, Sequencing, and Bioinformatics

Genomic DNA was extracted from all selected ESBL-producing *Enterobacteriaceae* and transconjugants using the commercial GenElute^TM^ Bacterial Genomic DNA Kit (Sigma-Aldrich) according to the manufacturer’s instructions. Genomic DNA was sequenced at Eurofins Genomics (Konstanz, Germany) on an Illumina HiSeq4000 instrument (paired-end, 2 × 150 bp). Raw data were assembled to contigs using CLC Genomics Workbench 10.0 (Qiagen, Venlo, Netherlands) for further analysis. The Illumina raw reads (ERR4065331–ERR4065341; ERR4065362–ERR4065369; and ERR4065372–ERR4065376) were submitted to the European Nucleotide Archive (ENA) under BioProject PRJEB37967. Additionally, plasmid DNA from ESBL-producing *E. coli* and their transconjugants was enriched using a commercial PureYield Plasmid Maxiprep System (Promega, Fitchburg, WI, USA). Enriched plasmid DNA was sequenced on a Pacific Biosciences RSII instrument and used for hybrid assemblies as described previously [15]. The PacBio raw reads (ERR4235917–ERR4235921; ERR4234695; ERR4235916) were also submitted to ENA under PRJEB37967.

In order to estimate the diversity of the tested strains, multi-locus sequence typing (MLST) was performed and a phylogenetic tree constructed from the assembled contigs, using the Center for Genomic Epidemiology (CGE) online tools MLST 2.0 and CSI Phylogeny 1.4 [33,34]. Phylogenetic trees were illustrated using FigTree v1.4.4 [35]. Antibiotic resistance genes and plasmid replicons were identified using ResFinder 3.2 and PlasmidFinder 2.1 [36,37]. All CGE tools were run with default settings for the respective tool version. One type strain per ESBL-producing species was included in the analysis (*C. freundii* ATCC8090, *E. cloacae* ATCC13047, and *E. coli* ATCC11775).

### 2.5. Plasmid Transfer Assays

To examine the frequency of ESBL resistance genes and phenotype transfer to non-ESBL-producers, ESBL-producing irrigation water isolates were used as donors in filter and broth conjugation assays. The donor strain collection comprised 11 ESBL *E. coli*, 5 ESBL *E. cloacae* complex, and 3 ESBL *C. freundii* complex strains. *Escherichia coli* CV601 served as a recipient strain in all experiments.

The filter conjugation assay was adapted from a previously described method [38]. Donor and recipient strains were grown overnight in Tryptic Soy Broth (TSB, Merck, Darmstadt, Germany) supplemented with CAZ (8 or 0.8 mg/L), RIF (50 mg/L), and KAN (50 mg/L), respectively. Absorbance was measured and adjusted to OD_600_ = 4.0 ± 0.5 for all cultures. Recipient and donor cultures were centrifuged (3100 × *g*, 5 min), the supernatants discarded, the pellets washed twice with 1 mL of 0.1 × TSB, and finally re-suspended in 1 mL of 0.1 × TSB. Then, 500 µL of each donor culture was added to 500 µL of recipient culture and mixed by pipetting. The resulting conjugation mixture was centrifuged (3100 × *g*, 5 min), the supernatant discarded, and the pellet re-suspended in 200 µL of 0.1 × TSB. For conjugation, Durapore^®^ membrane filters (Merck Millipore, Burlington, MA, USA) were placed on Plate Count Agar (PCA; Thermo Fisher Scientific, Waltham, MA, USA), and the entire conjugation mixture was transferred to the center of the filter. As controls, after washing, 1 mL of each donor culture only or recipient culture only was centrifuged, re-suspended in 200 µL of 0.1 × TSB, and transferred to a filter. After overnight incubation at 28 °C, filters were placed in 2 mL of sterile 0.9% NaCl solution and vortexed for 1 min. Conjugation suspensions and controls were serially diluted and appropriate dilutions plated in duplicates on PCA plates containing RIF (50 mg/L), KAN (50 mg/L), and CAZ (8 or 0.8 mg/L). To determine the number of recipient cells, spot plating was performed for each conjugation mixture by spotting three 20 µL drops per dilution (10^−5^ to 10^−8^) on PCA supplemented with RIF (50 mg/L) and KAN (50 mg/L). To determine transfer frequencies, the total number of transconjugants was divided by the number of recipients as determined by spot plating. All plates were incubated at 28 °C for up to 72 h.

The broth conjugation assay was performed as described previously [38]. In short, donor and recipient strains were grown in LB overnight (37 °C, 180 rpm). In 2 mL Eppendorf tubes containing 1 mL of LB, 500 µL of each donor strain were mixed with 500 µL of recipient strain and incubated for 24 h at 37 °C. Conjugation mixtures were serially diluted, and 100 µL of appropriate dilutions were plated in duplicate onto LB agar plates containing KAN (50 mg/L), RIF (50 mg/L), and CAZ (8 or 0.8 mg/L) followed by incubation at 37 °C for up to 48 h.

For both, filter and broth conjugation assays, green fluorescence was confirmed under UV light for putative transconjugants. Per replicate, three transconjugants were picked and re-streaked. For confirmation of *E. coli* CV601 background in transconjugants, repetitive sequence-based PCR fingerprinting (REP-PCR) was performed using the (GTG)_5_ primer (5′-GTGGTGGTGGTGGTG-3′) [39]. Template DNA of putative transconjugants and recipient was obtained by suspending a single colony in 50 µL of sterile water followed by boiling for 10 min at 99 °C. For PCR conditions see Table A1.

## 3. Results

### 3.1. Phylogenetic Diversity of Donor Strains

**Figure 1:**
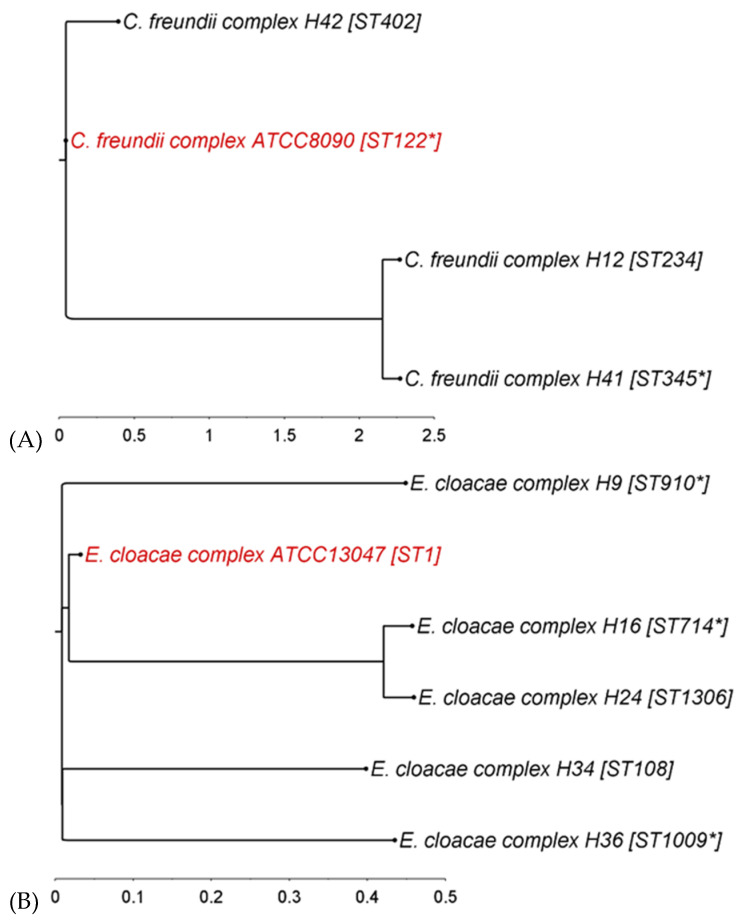

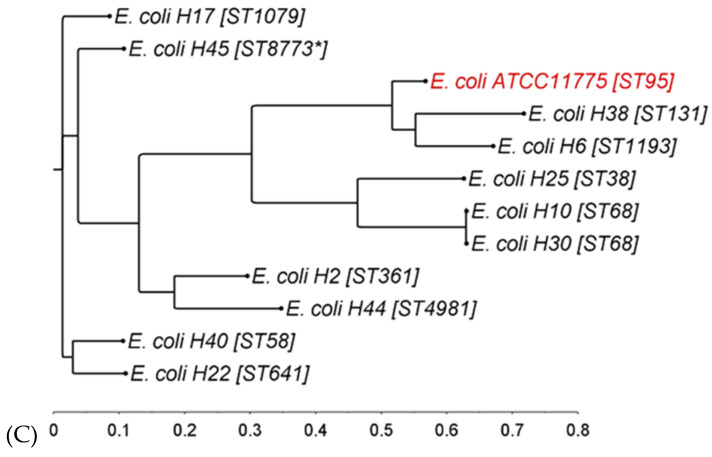
Phylogenetic trees of ESBL-producing (**A**) *C. freundii* complex, (**B**) *E. cloacae* complex, and (**C**) *E. coli* strains based on single-nucleotide polymorphisms. Reference strains in red. ST, sequence type; for strains with unknown ST, the nearest known ST is marked by an asterisk (*) (illustration: FigTree v1.4.4).

The phylogenetic diversity of the ESBL-producing strains was analyzed by MLST typing and by calculating single-nucleotide polymorphism (SNP)-based phylogenetic trees (Figure 1).

For *C. freundii* complex strains, 78.5% of the reference type strain genome was covered by all three isolates, with the individual strains sharing at least 80.6% with the reference. Two strains (H12 and H41) clustered together in one clade, while the third strain (H42) was separated from them by the reference strain (Figure 1A). All four strains belonged to different sequence types (STs).

In the *E. cloacae* complex, 64.7% of the reference strain genome was covered by all five irrigation water *E. cloacae* strains and at least 71.1% by the single strains. Strains H16 and H24 clustered closely in one clade, while the others were separated clearly, with the reference strain placed in between them. They all belonged to different STs, while three belonged to unknown sequence types for which the nearest known ST is indicated (Figure 1B).

All together, the analyzed ESBL-producing *E. coli* covered 72.6% of the reference strain genome with a minimum of 80.2% shared genome when comparing individual strains to the reference. Two strains (H10 and H30) belonged to ST68 and consequently clustered together in the tree (Figure 1C). Strain H38, belonging to the global pandemic clone ST131, closely clustered with strain H6 (ST1193) and the reference strain. Taken together, the ESBL-producing *E. coli* used in this study represented a broad diversity of STs, with the reference type strain lying in between them (Figure 1C). Interestingly, three strains originating from the same water source (H2, H6, and H17) did not cluster together in the tree, demonstrating that irrigation water can contain multiple and distantly related ESBL-producing *E. coli*. On the other hand, H10 and H25, also originating from one irrigation water sample, clustered closely together, although they belonged to different STs.

Finally, SNP-distance matrices were calculated for each species group, featuring the number of SNPs detected by pairwise comparison. Based on the minimum and maximum SNP differences detected, *E. coli* showed the largest range from nearest to farthest strain (min. 25; max. 46,239), followed by *E. cloacae* (min. 6913; max. 52,453) and *C. freundii* (min. 13,962; max. 66,505). In terms of maximum SNP difference, the *C. freundii* strains were the most dispersed.

### 3.2. Antibiotic Resistance and Plasmid Profiles of Donor Strains

For the ESBL-producing *E. coli* from different irrigation waters, phenotypic resistance determined by disk diffusion as well as ARGs and plasmid replicons detected by sequencing have been published previously [15]. Briefly, the ARGs responsible for the observed ESBL phenotype all belonged to the *bla*_CTX-M_ gene family. The most frequently detected were *bla*_CTX-M-1_ (4 of 11) and *bla*_CTX-M-15_ (3 of 11). Five of the 11 detected *bla*_CTX-M_ genes were linked to a known plasmid replicon, 3 of which were identified as IncI1.

Disk diffusion profiles as well as ARGs and plasmid replicons detected in ESBL-producing *E. cloacae* and *C. freundii* are shown in Table 1. In four of five *E. cloacae* strains, β-lactam resistance genes of the AmpC-type were detected (*bla*_ACT_), while no β-lactamase gene was detected in one *E. cloacae* strain. In *C. freundii* strains as well, β-lactamases of Ambler class C were detected (*bla*_CMY_ genes). Many of these *bla*_CMY-2_-like genes occur naturally in *C. freundii*, among others the detected *bla*_CMY-48_ and *bla*_CMY-83_ [40]. Additionally, in *C. freundii* H42 a *bla*_TEM-1B_ gene was detected. Apart from ARGs encoding β-lactamases, ARGs associated with resistance to fosfomycin (*fosA* genes), colistin (*mcr-9*), and the quinolone ciprofloxacin (*qnrE1* and *qnrB*) were detected. Again, *C. freundii* H42 harbored additional ARGs in good correspondence to its observed richer phenotypic resistance profile (Table 1).

### 3.3. Filter and Broth Conjugation

**Figure 2:**
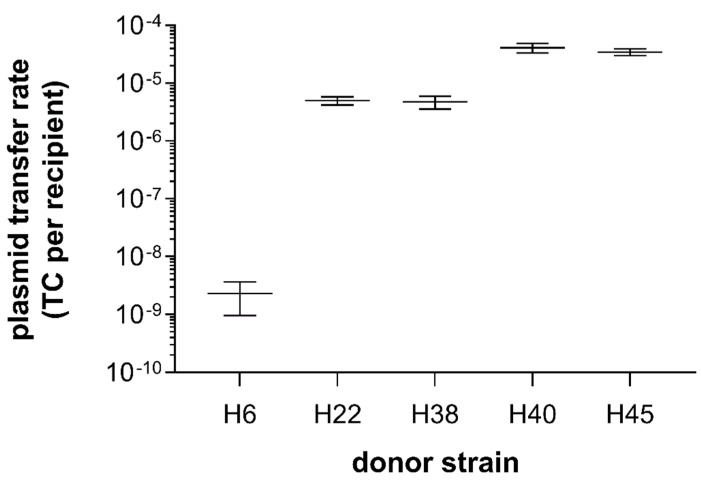
Plasmid transfer rates from ESBL-producing *E. coli* donor strains to a susceptible recipient strain (*E. coli* CV601) in filter conjugation assays (*n* = 3). Transconjugant (TC) numbers are normalized by the number of recipient bacteria per assay. Mean and standard error (SEM) are shown.

From the 19 ESBL-producing *Enterobacteriaceae* strains used in the filter conjugation assay, only 5 *E. coli* strains (for H6, H22, H38, H40, and H45) could transfer their resistance to a non-ESBL-producing recipient strain, resulting in ESBL-producing transconjugants (Figure 2). In the broth conjugation assay, these same five strains were the only ones to transfer successfully the ESBL-phenotype to the recipient strain. The successful transfer was confirmed by the matching REP-PCR fingerprint profiles of the transconjugants and the recipient. Plasmid transfer frequencies in filter conjugation assays were 2.3 × 10^−9^ (± 1.9 × 10^−9^) for H6, 5 × 10^−6^ (± 1.1 × 10^−6^) for H22, 4.7 × 10^−6^ (± 1.7 × 10^−6^) for H38, 4.1 × 10^−5^ (± 1.1 × 10^−5^) for H40, and 3.5 × 10^−5^ (± 6.8 × 10^−6^) for H45, respectively (Figure 2). Overall, conjugation rates were very similar between biological replications, with the exception of H6, whose conjugation rates were close to the detection limit. Of note, strain H6 harbors the *bla*_CTX-M_ gene with the most associated ARGs, summing up to a total of nine ARGs on one contig (see below).

### 3.4. Donor and Transconjugant Strain Comparison

#### 3.4.1. Phenotypic Antibiotic Resistance

Broth microdilution results from ESBL-producing *E. coli* and their respective transconjugants are shown in Table 2. No resistance was observed for any strain towards cefoxitin, chloramphenicol, colistin, doripenem, ertapenem, fosfomycin, gentamycin, imipenem, meropenem, piperacillin–tazobactam, and tigecycline.

The recipient strain *E. coli* CV601 showed intermediate resistance towards amikacin (AMK) and tobramycin (TOB), as expected due to presence of a *gfp*-*aph(3′)-III* cassette. These two resistances were therefore detected in most transconjugants as well (Table 2). Most importantly, the ESBL phenotype was detected phenotypically in all transconjugants. Resistance phenotypes of H6 and H6-TC were identical with the exception of fluoroquinolone resistance (ciprofloxacin (CIP) and levofloxacin (LVX)) observed in the donor strain only. Strains H22 and H22-TC shared all phenotypic resistances, with the exception of recipient-associated aminoglycoside resistance (Table 2). Strain H38 and the respective transconjugant strain were identical in their resistances towards β-lactams. They differed in H38 having fluoroquinolone resistances (CIP and LVX; Table 2). Comparing H40 to its transconjugant, we again observed a common β-lactam resistance profile. In terms of differences, H40 showed resistance to trimethoprim–sulfamethoxazole (SXT), minocycline (MI), and tetracycline (TE), while H40-TC to TE only (and one in three replicates resistant towards MI). Finally, H45 and H45-TC shared all β-lactam resistances.

#### 3.4.2. Molecular Detection of ARGs and Resistance Point Mutations

On the molecular side, we identified ARGs, plasmid replicons, and point mutations known to confer antibiotic resistance in transconjugant strains and their respective ESBL-producing donors. The detected ARGs grouped by contig are in complete accordance between donor and respective transconjugant strain for all five donor-transconjugant pairs (Table 3). Each of the contigs carrying the *bla*_CTX-M_ gene was transferred as an entity to the recipient strain, including the long ARG contig of strain H6 encompassing nine ARGs in total. Moreover, the plasmid replicons associated with the *bla*_CTX-M_ contigs in donor strains were detected in the transconjugant strains as well, with H6-TC forming the only exception. Finally, all transconjugants contained *aph(3′)-III* originating from the recipient strain.

Strain H40 was the only donor to harbor a second, non-ESBL plasmid of the IncFIB/IncFIC replicon family with seven ARGs (Table 3). However, transfer to the recipient strain was not detected—as opposed to the IncI1-associated *bla*_CTX-M-1_*, sul2,* and *tet(A)* genes—since the plasmid did not carry ESBL genes; thus, transconjugants were not selected.

Several point mutations conferring resistance to fluoroquinolones were identified in the donors H6 and H38. These included mutations in the A-subunit of the DNA gyrase as well as in the genes *parC* and *parE*, encoding subunits of the DNA topoisomerase IV (Table 3). No point mutations were detected in any of the transconjugant strains.

## 4. Discussion

Bacterial MLST can be used in source tracking. As opposed to extensive *E. coli* isolate databases revealing associations between known STs and strain origin, information about *E. cloacae* and *C. freundii* isolates is scarcer. As described in our previous work [15], the ESBL-producing *E. coli* STs detected in Swiss irrigation water have been detected previously in water and sewage, but also in humans, domesticated and wild animals, and different food items. As for *C. freundii*, the corresponding public MLST database [42] contains one entry for a ST234 strain from a diarrheal patient in China and one record for ST345 isolated from food in China. No records exist to date for *C. freundii* ST402. *Enterobacter cloacae* ST108 seems to be among the globally most widespread STs [43]. Additionally, three major lineages have been identified among MDR *E. cloacae*, one of which was defined as ST108-like [44]. Finally, *E. cloacae* ST910 has been associated with carbapenemase-carrying *E. cloacae* from sewage water of different Chinese hospitals [45] as well as German surface water [46]. Overall, the diversity and geographic distance of the different STs that were isolated from these sources emphasize the global distribution of these strains.

No transfer of ESBL phenotype or genes could be observed for any of the investigated *E. cloacae* or *C. freundii* strains, although known ESBL genes were detected in all but one isolate. However, no plasmid replicons could be identified, suggesting that these genes were not plasmid-borne. The *bla*_CMY-2_-like genes detected in all three *C. freundii* strains have been identified on plasmids previously [47]. However, they are also known to occur naturally in this species (that is, to confer intrinsic resistance), and could have been mobilized from the *C. freundii* chromosome onto plasmids by mediation of the insertion sequence IS*Ecp1* [47]. In *E. cloacae* strains *bla*_ACT_ genes were detected. Transmission of such genes between bacteria has also been described [48]. However, in *Enterobacter* spp. they are known to be intrinsic [49], making them unable of transferring to other bacteria unless gene mobilization takes place first.

Resistance phenotype and genotype of *C. freundii* and *E. cloacae* were in good accordance (Table 1). The ESBL phenotype relied on the presence of the above-mentioned AmpC-type ARGs. The presence of *qnr* genes was accompanied by phenotypic quinolone resistance. The colistin ARG *mcr-9* has been documented to not always confer phenotypic resistance [40], and accordingly we did not observe any colistin resistance phenotypically. Finally, the resistance phenotype of *C. freundii* H42 nicely reflected all ARGs detected, including tetracycline, sulfonamide, and trimethoprim resistance. Additionally, a plasmid replicon was detected in this strain that was not, however, associated with the *bla*_CMY_ gene after assembly, but with *sul2* only.

Conjugation experiments were conducted on filter/solid medium as well as in liquid broth cultures, as conjugation success can vary between the two systems. We did not observe, however, a difference as the same strains conjugated under both conditions. The five *E. coli* strains able to transfer their ESBL phenotype by conjugation were the same five in which a known plasmid replicon was associated with the *bla*_CTX-M_ gene. Plasmid-mediated transmission was further confirmed by the detection of all donor plasmid replicons associated with the *bla*_CTX-M_ genes in the transconjugants, except for H6-TC. This latter displayed the lowest conjugation rates while harboring the most ARG on the *bla*_CTX-M_ contig. This heavy ARG burden possibly associated with a large plasmid might be one reason for the low conjugation rates.

The phenotypic resistance profiles of donor and respective TC strains observed in broth microdilution are in good accordance with the molecular resistance profiles. Their different genetic backgrounds can explain most differences between donor and respective transconjugant strains. First, we observed intermediate to full resistance towards the tested aminoglycosides amikacin and tobramycin in most transconjugants. This resistance originates from the recipient strain *E. coli* CV601, which carries *aph(3′)-III* encoded by a *gfp*-cassette and conferring resistance to kanamycin and other aminoglycosides. On the other hand, the donors H6 and H38 were resistant to fluoroquinolones (CIP and LVX), which rely on point mutations in the respective strains and cannot transfer to the recipient. Finally, the H40-TC lost SXT resistance as compared to H40, since *dfrA1* for trimethoprim resistance was located on the second, non-ESBL plasmid of this donor strain. Tetracycline resistance, however, transferred along with *bla*_CTX-M-1_ and was observed phenotypically in both H40 and H40-TC.

In conclusion, none of the investigated ESBL-producing *E. cloacae* and *C. freundii* strains transferred ESBL-encoding genes by conjugation, due to the intrinsic nature of these ARGs. On the other hand, we could show significant conjugative transfer of ESBL-encoding MDR plasmids from irrigation water-borne ESBL-producing *E. coli* to ESBL-susceptible *E. coli*, with conjugation in 5 out of 11 strains (up to 4.1 × 10^−5^ TC per recipient bacterium). These results highlight the importance of monitoring irrigation water quality for selected ARB in order to mitigate contamination of the irrigated produce with clinically significant bacteria, whose MDR plasmids can moreover spread in bacterial communities.

## Figures and Tables

**Table 1:** Antibiotic resistance phenotype, resistance genes, and plasmid replicons in ESBL-producing *E. cloacae* and *C. freundii* complex strains from irrigation water. Full phenotypic resistance (R) shown in bold, intermediate phenotypic resistance (I) in plain font; % ID, percent identical bases between query and sample sequence; HSP/query, alignment length of sample to query sequence. AM: ampicillin; TEMO: temocillin; AMC: amoxicillin–clavulanic acid; TPZ: piperacillin–tazobactam; KF: cefalotin; FOX: cefoxitin; CXM: cefuroxime; CPD: cefpodoxime; CRO: ceftriaxone; CTX: cefotaxime; CAZ: ceftazidime; FEP: cefepime; ETP: ertapenem; TE: tetracycline; MI: minocycline; NA: nalidixic acid; TMP: trimethoprim; SMZ: sulfonamide; SXT: trimethoprim–sulfamethoxazole; F100: nitrofurantoin.

Strain ID	Antibiotic Resistance Phenotypes (R and I)	Acquired Antibiotic Resistance Genes	Plasmid Replicons (% ID; HSP/Query)
Ent.H9	**AM, TEMO, AMC, TPZ, KF, FOX, CXM, CPD, CRO, CTX, CAZ, ETP, SMZ, SXT**	*bla*_ACT-9_, *fosA*†, *mcr-9*	none detected
Ent.H16	**AM, TEMO, AMC, TPZ, KF, FOX, CXM, CPD, CRO, CTX, CAZ, ETP, F100**	*bla*_ACT-12_*, fosA2*†	none detected
Ent.H24	**AM, TEMO, AMC, TPZ, KF, FOX, CXM, CPD, CRO, CTX, CAZ, ETP, F100**	*bla*_ACT-12_*, fosA2*†	none detected
Ent.H34	**AM, TEMO, AMC, TPZ, KF, FOX, CXM, CPD, CRO, CTX, CAZ, ETP, F100**	*bla*_ACT-7_	none detected
Ent.H36	**AM, TEMO, AMC, TPZ, KF, FOX, CXM, CPD, CRO, CTX, CAZ, ETP, MI, NA, F100**	*fosA*†*, qnrE1*	none detected
Citr.H12	**AM, TEMO, AMC, TPZ, KF, FOX, CXM, CPD, CRO, CTX, CAZ, FEP, NA, F100**	*bla*_CMY-83_	none detected
Citr.H41	**AM, TEMO, AMC, TPZ, KF, FOX, CXM, CPD, CRO, CTX, CAZ, FEP, ETP**	*bla*_CMY-101_	none detected
Citr.H42	**AM, AMC, TPZ, KF, FOX, CXM, CPD, CRO, CTX, CAZ, TE, MI, NA, TMP, SMZ, SXT**	*bla*_TEM-1B_*, bla*_CMY-48_*, tet(B), qnrB, dfrA17, sul1, sul2, aadA5* ˟*, catA1* ˟	IncQ1 (100; 529/796) *

* truncated form of IncQ1, associated with *sul2*; ˟ resistance towards streptomycin and chloramphenicol was not tested in disk diffusion assays; † resistance towards fosfomycin cannot be determined reliably in disk diffusion assays [41].

**Table 2:** Antibiotic resistance phenotypes of recipient strains *E. coli* CV601, ESBL-producing *E. coli*, and their respective transconjugants (TCs) obtained from filter conjugation, determined using broth microdilution. S (sensitive), I (intermediate), and R (resistant) phenotypes are based on EUCAST cut-off values. +, ESBL confirmation; AMK, amikacin; AMC, amoxicillin–clavulanic acid; SAM, ampicillin–sulbactam; AM, ampicillin; ATM, aztreonam; FEP, cefepime; CTX, cefotaxime; CAZ, ceftazidime; CXM, cefuroxime; CIP, ciprofloxacin; LVX, levofloxacin; MI, minocycline; PIP, piperacillin; TE, tetracycline; TOB, tobramycin; SXT, trimethoprim–sulfamethoxazole.

	Antibiotic Resistant Phenotypes															
Strain ID	AMK	AMC	SAM	AM	ATM	FEP	CTX	CAZ	CXM	CIP	LVX	MI	PIP	TE	TOB	SXT
*E. coli* CV601	I	S	S	S	S	S	S	S	S	S	S	S	S	S	I	S
H6	S	S	R	R	+	R	+	+	R	R	R	S	R	R	S	R
H6-TC	S	S	R	R	+	R	+	+	R	S	S	S	R	R	S	R
H22	S	R	R	R	+	R	+	+	R	S	S	I	R	R	S	S
H22-TC	I	R	R	R	+	R	+	+	R	S	S	I	R	R	I	S
H38	S	S	R	R	+	R	+	+	R	R	R	S	R	S	S	S
H38-TC	I	S	R	R	+	R	+	+	R	S	S	S	R	S	S	S
H40	S	R	R	R	+	R	+	+	R	S	S	I	R	R	S	R
H40-TC	I	R	R	R	+	R	+	+	R	S	S	S	R	R	I	S
H45	S	R	R	R	+	R	+	+	R	S	S	S	R	S	S	S
H45-TC	R	R	R	R	+	R	+	+	R	S	S	S	R	S	R	S

**Table 3:** Antibiotic resistance genotype including plasmid replicons in ESBL-producing *E. coli* and their respective transconjugants obtained from filter conjugation. All detected plasmid replicons were full length.

ESBL-Producing Donors				Transconjugants			
ID	Acquired ARGs (Grouped by Contig)	Plasmid Replicons (% ID)	Point Mutations	ID	Acquired ARGs (Grouped by Contig)	Plasmid Replicons (% ID)	Point Mutations
H6	I. *strA, strB, aadA5, bla*_CTX-M-27_*, mph(A), sul1, sul2, tet(A), dfrA17*	I. IncFIA (99.74), IncFIB (96.63)	parE p.L416F parC p.S80I gyrA p.S83L gyrA p.D87N	H6-TC	I. *strA, strB, aadA5, bla*_CTX-M-27_*, mph(A), sul1, sul2, tet(A), dfrA17* II. *aph(3′)-III*	none detected	none detected
H22	I. *bla*_CTX-M-1_*, sul2, tet(A)*	I. IncI1 (98.59)	none detected	H22-TC	I. *bla*_CTX-M-1_*, sul2, tet(A)* II. *aph(3′)-III*	I. IncI1 (99.3)	none detected
H38	I. *bla*_CTX-M-15_*, mph(A)*	I. IncFIB (98.39)	parE p.I529L parC p.S80I parC p.E84V gyrA p.S83L gyrA p.D87N	H38-TC	I. *bla*_CTX-M-15_*, mph(A)* II. *aph(3′)-III*	I. IncFIB (98.39)	none detected
H40	I. *bla*_CTX-M-1_*, sul2, tet(A)* II. *strA, strB, aadA1, sul1, sul2, tet(A), dfrA1*	I. IncI1 (98.59) II. IncFIB (97.07), IncFIC (95.59)	none detected	H40-TC	I. *bla*_CTX-M-1_*, sul2, tet(A)* II. *aph(3′)-III*	I. IncI1 (98.59)	none detected
H45	I. *bla*_CTX-M-1_	I. IncI1 (98.59)	none detected	H45-TC	I. *bla*_CTX-M-1_ II. *aph(3′)-III*	I. IncI1 (99.3)	none detected

## Appendix A

**Table A1:** **Repetitive sequence-based PCR fingerprinting** (REP-PCR) cycling conditions. Used for confirming *E. coli* CV601 background in putative transconjugants using the (GTG)_5_ primer (5′-GTGGTGGTGGTGGTG-3). PCRs were performed using the HotStarTaq^®^ Master Mix Kit (Qiagen, Hilden, Germany) in 25 µL reaction volumes.

Cycling Step	PCR Conditions		
Enzyme activation	95 °C, 5 min		
Denaturation	94 °C, 3 s		
	92 °C, 30 s		30 cycles
Annealing	40 °C, 1 min		
Extension	65 °C, 8 min		
Final extension	65 °C, 16 min		
